# An evaluation of piperacillin-tazobactam antimicrobial stewardship intervention and association with β-lactam susceptibility in *Pseudomonas aeruginosa* and *Enterobacter cloacae* complex

**DOI:** 10.1017/ash.2026.10911

**Published:** 2026-10-16

**Authors:** Ivayla I. Geneva, Kayode Medugbon, Stephen J. Thomas, Andrea Call

**Affiliations:** 1 Crouse Hospital, Syracuse, NY, USA; 2 State University of New York Upstate Medical University, Syracuse, NY, USA

## Abstract

**Introduction:**

Antimicrobial resistance is a growing global concern. At our hospital, susceptibility of Pseudomonas aeruginosa to piperacillin-tazobactam declined from 95% to 77%, while susceptibility of Enterobacter cloacae complex decreased from 82% to 72%, prompting antimicrobial stewardship program (ASP) interventions.

**Methods:**

ASP interventions aimed to reduce unnecessary antipseudomonal therapy with ceftriaxone recommended for low-risk patients, to replace piperacillin-tazobactam with cefepime in high-risk patients. Additionally, pharmacodynamic optimization was introduced for piperacillin-tazobactam, cefepime, and ceftriaxone. Annual inpatient antibiograms and antibiotic use (days of therapy per 1,000 patient-days) were evaluated for piperacillin-tazobactam, cefepime, meropenem, ciprofloxacin, levofloxacin, and ceftriaxone. Spearman correlation assessed the relationship between antibiotic use and bacterial susceptibility with a 1-year lag.

**Results:**

Over the two years following the initiation of interventions in 2023, piperacillin-tazobactam use decreased 11-fold, cefepime use increased 2.6-fold, and ceftriaxone use increased by 1.7-fold. Meropenem use declined 1.5-fold. P. aeruginosa susceptibility improved: piperacillin-tazobactam (77% to 96%), meropenem (83% to 92%), and cefepime (77% to 96%). E. cloacae complex susceptibility to piperacillin-tazobactam increased from 63% to 86%. Piperacillin-tazobactam use demonstrated a statistically significant perfect negative correlation with P. aeruginosa susceptibility. Cefepime use showed a strong positive, non-significant correlation with P. aeruginosa susceptibility, whereas the correlation between piperacillin-tazobactam use and E. cloacae susceptibility was moderately negative but not significant.

**Conclusions:**

ASP-driven restriction of piperacillin-tazobactam was associated with marked improvement in P. aeruginosa susceptibility to both piperacillin-tazobactam and cefepime despite substantially increased cefepime use, suggesting shared resistance mechanisms and that piperacillin-tazobactam may be a major driver of resistance selection.

## Introduction

Antimicrobial resistance (AMR) has emerged as one of the most significant threats to global public health, reducing the effectiveness of antimicrobial therapies, increasing morbidity and mortality, prolonging hospitalizations, escalating healthcare expenditures, and jeopardizing advances in modern medicine.^1,2^ Recent estimates suggest that bacterial AMR contributes to more than one million deaths annually worldwide and is associated with several million additional deaths each year.^1^ In response to the escalating burden of resistant infections, the World Health Organization updated its Bacterial Priority Pathogens List in 2024, identifying resistant Gram-negative organisms—including *Pseudomonas aeruginosa* and resistant Enterobacterales (such as *Enterobacter cloacae, Escherichia coli, and Klebsiella pneumoniae*, and many others)—as among the most urgent threats requiring novel therapeutic strategies and intensified surveillance efforts.^3^

Piperacillin-tazobactam and cefepime remain among the most commonly used antipseudomonal β-lactams. Both agents are frequently utilized as carbapenem-sparing options within antimicrobial stewardship programs (ASPs), including at our hospital, because of their broad activity against *P. aeruginosa* and the Enterobacterales.^4^ However, increasing resistance to these agents has threatened their effectiveness. Previous studies have demonstrated that antimicrobial consumption is an important driver of resistance selection and that reductions in the use can be associated with subsequent improvements in susceptibility patterns^5,6^

Although several studies have evaluated the relationship between antimicrobial use and resistance at the population level, the impact of targeted stewardship interventions on susceptibility patterns among *P. aeruginosa* and *E. cloacae* complex remains incompletely understood. In particular, the relative contribution of piperacillin-tazobactam exposure to resistance selection has not been fully elucidated. Because many resistance mechanisms affecting piperacillin-tazobactam also influence susceptibility to other β-lactams, changes in the use of a single agent may have broader ecological effects on institutional antibiograms than would be expected based solely on direct antibiotic exposure. Understanding these relationships is increasingly important as stewardship programs seek to balance effective empiric therapy with preservation of antimicrobial activity via antibiotic use restriction.

In this paper, we provide a description of the response of our hospital’s ASP to a marked decline in susceptibility of both *P. aeruginosa* and *E. cloacae* complex to piperacillin-tazobactam. We hypothesized that reducing piperacillin-tazobactam utilization will lead to improved susceptibility among *P. aeruginosa* and *E. cloacae* complex and sought to quantitatively evaluate the longitudinal relationships between institutional antimicrobial use and susceptibility patterns to several additional antibiotic classes—cephalosporins, carbapenems, and fluoroquinolones.

## Methods

### Antimicrobial stewardship interventions for antipseudomonal agents

This is a prospective interventional study carried out at a tertiary medical center (Crouse Hospital in Syracuse, NY). A sharp decrease was noted in *P. aeruginosa*’s (95% to 77%) and *E. cloacae* complex’ (82% to 72%) susceptibility to piperacillin-tazobactam, based on our hospital’s inpatient antibiogram that reports data annually. To address this problem, ASP interventions were initiated in April 2023 and carried out as detailed in Table 1. We aimed to minimize the use of empiric antipseudomonal coverage in low-risk cases, where “low risk” was defined as patients lacking the following recognized epidemiologic and clinical risk factors: no prior isolation or colonization with *P. aeruginosa* within the preceding 12 months, no receipt of intravenous broad-spectrum antibiotics within the previous 90 days, no hospitalization exceeding 48 hours within the previous 90 days, no residence in a long-term acute care hospital or skilled nursing facility with known high prevalence of multidrug-resistant Gram-negative organisms, no structural lung disease associated with chronic Pseudomonas colonization such as bronchiectasis, cystic fibrosis, advanced chronic obstructive pulmonary disease with frequent exacerbations, no severe immunocompromising condition, including prolonged neutropenia, hematopoietic stem cell transplantation, or recent solid organ transplantation, no healthcare-associated pneumonia, ventilator-associated pneumonia, or severe hospital-acquired infection, no septic shock or other critical illness requiring intensive care unit admission at presentation, no local microbiologic data suggesting a high likelihood of *P. aeruginosa* involvement for the specific syndrome being treated.^4,7^ The ASP pharmacist referred to patients’ hospital charts with a particular focus on the history of present illness admission note to evaluate the above-listed risk factors. Further, a prominent note was placed in ordering set stating: “Piperacillin/Tazobactam is inadequate as empiric coverage for Enterobacter or Pseudomonas per Crouse 2023 Antibiogram.” Cefepime plus/minus Metronidazole (if anaerobic coverage was needed) was substituted for Piperacillin/Tazobactam as the first-choice antibiotic in the relevant order sets. Announcements regarding these changes and the new treatment recommendations were communicated to all inpatient providers at several timepoints as detailed in Table 1. Daily (Monday through Friday) screens by the ASP pharmacist of all Piperacillin/Tazobactam orders and in-person evaluation by an ID physician of selected cases to consider change to cefepime if empiric Pseudomonas coverage is needed or de-escalation to ceftriaxone if empiric Pseudomonas coverage is not needed, followed by direct reach out by the ASP pharmacist, over phone or in person, to prescribers to deliver the appropriate antibiotic recommendations. Most cases were addressed a single time by the ASP pharmacist with an average overall uptake of the ASP recommendations by the hospital providers being at or above 80%.

**Table 1.** Antimicrobial stewardship program interventions Table 1 long description.

Antimicrobial Stewardship Program (ASP) interventions
**April 2023** Piperacillin/tazobactam was changed to extended infusion dosing to enhance pharmacodynamic target attainment Cefepime was changed to 2 g from 1g standard dosing to prevent development of resistance A prominent note was placed in ordering set stating: “Piperacillin/Tazobactam is inadequate as empiric coverage for Enterobacter or Pseudomonas per Crouse 2023 Antibiogram” Cefepime + metronidazole was substituted for piperacillin/tazobactam as the first-choice antibiotic in the relevant order sets Susceptibility to cefepime was unhidden culture results An email was sent from the hospital Chief of Medicine to all physicians highlighting the concerning antibiogram results and the above changes Daily (Monday through Friday) screens by the ASP pharmacist of all piperacillin/tazobactam orders and in-person evaluation by an ID physician of selected cases to consider change to cefepime if empiric Pseudomonas coverage is needed or de-escalation to ceftriaxone if empiric Pseudomonas coverage is not needed, followed by direct reach out by the ASP pharmacist, over phone or in person, to prescribers to deliver the appropriate antibiotic recommendations—this ASP activity continues to date ASP pharmacist presented the above recommendations in PowerPoint format at the monthly hosiptalists’ meeting
**May 2023** Ninety paper notices were posted next to most hospital computer terminals summarizing the piperacillin/tazobactam to cefepime change for empiric Pseudomonas coverage
**June 2023** The ASP pharmacist sent FYI emails to all hospitalists and mid-level providers highlighting the above-listed recommendations regarding the preferential use of cefepime (plus metronidazole if anaerobic coverage needed) over Zosyn in suspected Pseudomonas infections and the preferred use of ceftriaxone over cefepime for cases without risk for Pseudomonas as well as a new recommendations for a higher dose of ceftriaxone (2g instead of the prior 1g routine use) in order to minimize evolution of resistance with all order sets updated to reflect the same The ASP pharmacist presented the above recommendations in PowerPoint format at the monthly physician assistants’ meeting The ASP pharmacist initiated daily (Monday through Friday) screens of all ceftriaxone orders followed by discussion with prescribers to encourage adherence to the preferred 2g dosing regimen—this ASP activity continues to date
**September 2023** The ASP pharmacist presented the above recommendations in PowerPoint format at the monthly nurse practitioners’ meeting

### Pharmacodynamic optimization for anti-Pseudomonal agents and other relevant antibiotics

Pharmacodynamic optimization measures at the beginning of this study included implementation of extended-infusion piperacillin-tazobactam dosing,^8^ increasing the standard cefepime dose from 1 g to 2 g,^9^ increasing the standard ceftriaxone dose from 1 g to 2 g,^10,11^ Finally, in October 2024 all inpatient infusions of cefepime were transitioned from the 30-minute standard infusions to extended infusions over 3 to 4 hours.^9,12^ Electronic order sets were modified to implement the above changes. Real-time pharmacist-driven oversight ensured that the appropriate dosing regimen were followed, with direct phone calls made to providers if a deviation from the recommendation was identified.

### Statistical analyses

Annual antibiograms based on inpatient isolates and antibiotic utilization (days of therapy per 1,000 patient-days) were measured for piperacillin-tazobactam, cefepime, meropenem, ciprofloxacin, levofloxacin, and ceftriaxone. Because changes in antimicrobial consumption may not be reflected immediately in institutional susceptibility patterns, correlations between antibiotic utilization and susceptibility were evaluated using a 1-year lag. This is consistent with prior ecological studies demonstrating delayed effects of antimicrobial selective pressure on resistance trends.^13,14^ Spearman’s rank correlation coefficient was used to assess associations between antibiotic use and subsequent-year susceptibility.

## Results

### Changes in antimicrobial utilization

**Figure 1.**
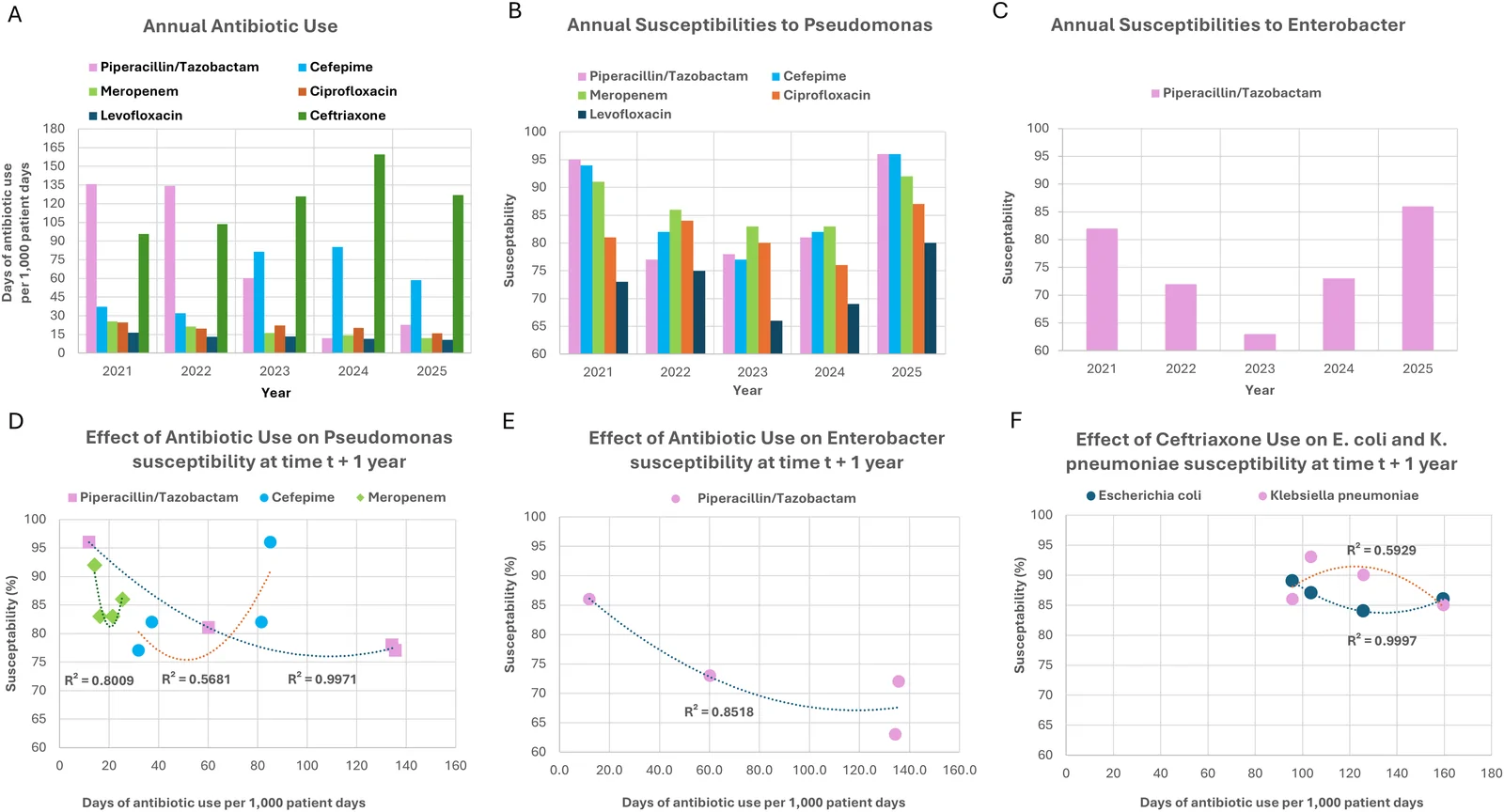
Antibiotic utilization and bacterial susceptibility for the period 2021–2025. (A) annual days antibiotic use per 1,000 patient days from 2021 to 2025 for piperacillin/tazobactam, cefepime, meropenem, ciprofloxacin, levofloxacin, and ceftriaxone; (B) annual *Pseudomonas aeruginosa* susceptibility to piperacillin/tazobactam, cefepime, meropenem, ciprofloxacin, and levofloxacin from 2021 to 2025; (C) annual *Enterobacter cloacae* complex susceptibility to piperacillin/tazobactam from 2021 to 2025; (D) scatterplot of annual antibiotic use versus annual *Pseudomonas aeruginosa* susceptibility to piperacillin/tazobactam showing perfect negative correlation, to cefepime showing strong positive correlation, and to meropenem showing strong negative correlation, none reaching statistical significance; (E) scatterplot of annual piperacillin/tazobactam use versus annual *Enterobacter cloacae* complex susceptibility to piperacillin/tazobactam showing a moderate negative correlation, not reaching statistical significance; (F) scatterplot of annual ceftriaxone use versus annual *Escherichia coli and Klebsiella pneumoniae* susceptibility to ceftriaxone showing strong and moderate negative correlation, respectively, but none reaching statistical significance. All trend lines were for polynomial fits, which yielded the highest R^2^ values.

Implementation of the antimicrobial stewardship intervention in April 2023 was associated with substantial changes in institutional antimicrobial utilization (Table 2, Figure 1A). Piperacillin-tazobactam use declined from 134.3 days of therapy (DOT) per 1,000 patient-days in 2022 to 60.2 in 2023 and reached a nadir of 12.0 in 2024, representing an 11.2-fold reduction compared with baseline, with slight increase to 22.8 DOT/1,000 patient-days in 2025. In contrast, cefepime utilization rose from 32.0 DOT/1,000 patient-days in 2022 to 81.5 s in 2023 and 85.2 in 2024 before declining to 58.8 in 2025 (Figure 1A). The peak cefepime utilization in 2024 represented a 2.7-fold increase relative to baseline. The use of ceftriaxone also increased from a baseline of 95.8 DOT/1,000 patient-days in 2021 to a maximum of 159.6 in 2024. Meropenem use declined progressively from 21.5 DOT/1,000 patient-days in 2022 to 12.2 in 2025, while levofloxacin utilization decreased from 13.1 to 10.8 DOT/1,000 patient-days during the same period. Ciprofloxacin use fluctuated modestly, increasing from 19.7 DOT/1,000 patient-days in 2022 to 22.2 in 2023 before declining to 15.9 in 2025.

**Table 2.** Annual antibiotic use and susceptibilities for *Pseudomonas aeruginosa*, *Enterobacter cloacae* complex, *Escherichia coli*, and *Klebsiella pneumoniae* for the period 2021–2025 Table 2 long description.

Antibiotic	Year	Total annual days of antibiotic use	Days of therapy per 1,000 patient days	*Pseudomonas aeruginosa* annual susceptibility based on data collected during the listed year
Piperacillin/tazobactam	2021	17,643	135.7	95% (N = 148)
	2022	17,292	134.3	77% (N = 114)
	2023	7,444	60.2	78% (N = 156)
	2024	1537	12.0	81% (N = 177)
	2025	2,951	22.8	96% (N = 142)
Cefepime	2021	4,854	37.3	94% (N = 148)
	2022	4,116	32.0	82% (N = 114)
	2023	10,081	81.5	77% (N = 156)
	2024	10,921	85.2	82% (N = 177)
	2025	7,592	58.8	96% (N = 142)
Meropenem	2021	3,304	25.4	91% (N = 148)
	2022	2,764	21.5	86% (N = 114)
	2023	2014	16.3	83% (N = 156)
	2024	1818	14.2	83% (N = 177)
	2025	1573	12.2	92% (N = 142)
Ciprofloxacin	2021	3,226	24.8	81% (N = 148)
	2022	2,535	19.7	84% (N = 114)
	2023	2,748	22.2	80% (N = 156)
	2024	2,601	20.3	76% (N = 177)
	2025	2,060	15.9	87% (N = 142)
Levofloxacin	2021	2,132	16.4	73% (N = 148)
	2022	1692	13.1	75% (N = 114)
	2023	1655	13.4	66% (N = 156)
	2024	1,484	11.6	69% (N = 177)
	2025	1,391	10.8	80% (N = 142)
Antibiotic	Year	Total annual days of antibiotic use	Days of therapy per 1,000 patient days	*Enterobacter cloacae* complex annual susceptibility based on data collected during the listed year
Piperacillin - Tazobactam	2021	17,643	135.7	82% (N = 45)
	2022	17,292	134.3	72% (N = 48)
	2023	7,444	60.2	63% (N = 47)
	2024	1537	12.0	73% (N = 42)
	2025	2,951	22.8	86% (N = 59)
Antibiotic	Year	Total annual days of antibiotic use	Days of therapy per 1,000 patient days	*Escherichia coli* annual susceptibility based on data collected during the listed year
Ceftriaxone	2021	12,455	95.8	90% (N = 361)
	2022	13,343	103.7	89% (N = 376)
	2023	15,554	125.8	87% (N = 395)
	2024	20,469	159.6	84% (N = 404)
	2025	16,409	127.0	86% (N = 382)
Antibiotic	Year	Total annual days of antibiotic use	Days of therapy per 1,000 patient days	*Klebsiella pneumoniae* annual susceptibility based on data collected during the listed year
Ceftriaxone	2021	12,455	95.8	89% (N = 134)
	2022	13,343	103.7	86% (N = 107)
	2023	15,554	125.8	93% (N = 101)
	2024	20,469	159.6	90% (N = 94)
	2025	16,409	127.0	85% (N = 96)

### Trends in P. aeruginosa susceptibility

*P. aeruginosa* susceptibility improved following implementation of the stewardship intervention, where the annual number of *P. aeruginosa* isolates included in susceptibility analyses ranged from 114 to 177 (Table 2, Figure 1B). Susceptibility to piperacillin-tazobactam had declined from 95% in 2021 to 77% in 2022 and remained low at 78% in 2023. Thereafter, susceptibility increased to 81% in 2024 and reached 96% in 2025. A similar pattern was observed for cefepime. Susceptibility decreased from 94% in 2021 to 82% in 2022 and further to 77% in 2023 before increasing to 82% in 2024 and 96% in 2025. Meropenem susceptibility remained relatively stable between 2023 and 2024 (83% in both years) before increasing to 92% in 2025. Ciprofloxacin susceptibility declined from 84% in 2022 to 76% in 2024 before rebounding to 87% in 2025. Likewise, levofloxacin susceptibility decreased from 75% in 2022 to 66% in 2023 before increasing to 80% in 2025.

### Trends in E. cloacae complex susceptibility

Improvements in susceptibility were also measured among *E. cloacae* complex isolates, with annual number of isolates ranging from 42 to 59 (Table 2, Figure 1C). Piperacillin-tazobactam susceptibility declined from 82% in 2021 to 72% in 2022 and further to 63% in 2023. Subsequently, susceptibility increased to 73% in 2024 and reached 86% in 2025, representing a 23% increase from the nadir observed in 2023 (Figure 1C).

### Trends in E. coli and K. pneumoniae susceptibility to ceftriaxone

As detailed in Table 2, there was a decrease in the susceptibility of *E. coli* to ceftriaxone from a baseline of 90% in 2021 to a nadir of 84% in 2024 and some improvement to 86% in 2025. Regarding *K. pneumoniae*’s susceptibility to ceftriaxone, there was some variation over the study period, from a baseline of 89% in 2021, decline to 86% in 2022, then rise to 93% in 2023 and then a 2-year decline to 90% in 2024 and to 85% in 2025.

### Correlation between antimicrobial utilization and subsequent-year susceptibility

Among *P. aeruginosa* isolates, piperacillin-tazobactam utilization demonstrated a statistically significant perfect negative correlation with subsequent-year piperacillin-tazobactam susceptibility (ρ = −1.00, *P* < .001) (Table 3, Figure 1D). Meropenem utilization also demonstrated a strong negative correlation with subsequent-year meropenem susceptibility (ρ = −0.80, *P* = .33), although statistical significance was not achieved. In contrast, cefepime utilization demonstrated a strong positive correlation with subsequent-year cefepime susceptibility (ρ = 0.85, *P* = .33) (Table 3, Figure 1D) without reaching statistical significance. Ciprofloxacin (ρ = −0.20, *P* = .80) and levofloxacin (ρ = −0.40, *P* = .60) demonstrated weak negative correlations with susceptibility.

**Table 3.** Spearman’s correlation analyses of the association between antibiotic use and the 1-year time-lagged susceptibility for *Pseudomonas aeruginosa and Enterobacter cloacae* complex Table 3 long description.

Antibiotic	Days of therapy per 1,000 patient days	*Pseudomonas aeruginosa* annual susceptibility at time t + 1 year	Spearman’s correlation (*ρ*)	*P* value (two-sided)	Interpretation
Piperacillin/tazobactam	135.7	77%	−1.00	0	***Perfect negative correlation***
	134.3	78%			
	60.2	81%			
	12.0	96%			
Cefepime	37.3	82%	0.85	.3	***Strong positive correlation*** Not statistically significant most likely due to the very small sample size (n = 4)
	32.0	77%			
	81.5	82%			
	85.2	96%			
Meropenem	25.4	86%	−0.80	.3	***Strong negative correlation*** Not statistically significant most likely due to the very small sample size (n = 4)
	21.5	83%			
	16.3	83%			
	14.2	92%			
Ciprofloxacin	24.8	84%	−0.20	.8	***No meaningful correlation***
	19.7	80%			
	22.2	76%			
	20.3	87%			
Levofloxacin	16.4	82%	−0.40	.6	***Weak negative correlation*** Not statistically significant
	13.1	88%			
	13.4	80%			
	11.6	92%			

Antibiotic	Days of therapy per 1,000 patient days	*Enterobacter cloacae* complex annual susceptibility at time t + 1 year	Spearman’s correlation (ρ)	*P* value (two-sided)	Interpretation
Piperacillin/tazobactam	135.7	72%	−0.40	.6	***Moderate negative correlation*** Not statistically significant most likely due to the very small sample size (n = 4)
	134.3	63%			
	60.2	73%			
	12.0	86%			
Cefepime	37.3	97%	−0.32	.7	**Weak negative correlation**
	32.0	99%			
	81.5	97%			
	85.2	97%			
Meropenem	25.4	99%	−0.89	.2	Given the susceptibility data’s extremely limited variability, the apparently strong negative relationship between meropenem use and Enterobacter susceptibility lagged by 1 year is ***unreliable.***
	21.5	99%			
	16.3	100%			
	14.2	100%			
Ertapenem	11.5	93%	0.11	.9	***No meaningful correlation***
	9.6	93%			
	8.3	93%			
	9.7	95%			
Ciprofloxacin	24.8	83%	−0.80	.3	***Strong negative correlation*** Not statistically significant most likely due to the very small sample size (n = 4)
	19.7	92%			
	22.2	85%			
	20.3	87%			
Levofloxacin	16.4	82%	−0.40	.6	***Weak negative correlation***
	13.1	88%			
	13.4	80%			
	11.6	92%			

Antibiotic	Days of therapy per 1,000 patient days	*Escherichia coli* annual susceptibility at time t + 1 year	Spearman’s correlation (ρ)	*P* value (two-sided)	Interpretation
Ceftriaxone	95.8	89%	−0.80	.2	***Strong negative correlation*** Not statistically significant most likely due to the very small sample size (n = 4)
	103.7	87%			
	125.8	84%			
	159.6	86%			

Antibiotic	Days of therapy per 1,000 patient days	*Klebsiella pneumoniae* annual susceptibility at time t + 1 year	Spearman’s correlation (ρ)	*P* value (two-sided)	Interpretation
Ceftriaxone	95.8	86%	−0.40	.6	***Moderate negative correlation*** Not statistically significant most likely due to the very small sample size (n = 4)
	103.7	93%			
	125.8	90%			
	159.6	85%			

Regarding the *E. cloacae* complex isolates, piperacillin-tazobactam utilization demonstrated a moderate negative correlation with subsequent-year susceptibility (ρ = −0.40, *P* = .60) (Table 3, Figure 1E), without reaching statistical significance. Similar weak-to-moderate negative correlations were observed for cefepime (ρ = −0.32, *P* = .68) and levofloxacin (ρ = −0.40, *P* = .60). Ciprofloxacin utilization demonstrated a stronger negative correlation with susceptibility (ρ = −0.80, *P* = .33). Meropenem utilization demonstrated a strong negative correlation (ρ = −0.89, *P* = .17) without statistical significance. No meaningful association was detected between ertapenem utilization and subsequent-year susceptibility (ρ = 0.11, *P* = .92).

Finally, as shown in Table 3, Figure 1F, we noted a strong negative correlation between the use of ceftriaxone and *E*. *coli*’s susceptibility to it (*ρ* =−0.8, *P* = .2). There was only a moderate negative correlation regarding *K*. *pneumoniae* (ρ =−0.4, *P* = .6). Neither reached statistical significance.

## Discussion

The relationship between antimicrobial consumption and resistance has been well established across multiple healthcare settings. Antibiotic exposure creates selective pressure favoring resistant organisms, and stewardship interventions designed to reduce unnecessary broad-spectrum antibiotic use have consistently been associated with reductions in AMR, Clostridioides difficile infection, and healthcare-associated infections.^15,16^ Our findings are consistent with these observations and provide additional evidence that targeted stewardship interventions may reverse unfavorable susceptibility trends even among highly adaptable pathogens such as *P. aeruginosa*.

Our study demonstrated how the implementation of a multifaceted antimicrobial stewardship intervention was associated with a major 11-fold (equivalent to 91%) reduction in piperacillin-tazobactam utilization and marked improvements in antimicrobial susceptibility among both *P. aeruginosa* and *E. cloacae* complex. Regarding the effect on Piperacillin-tazobactam use, ours is the highest ever reported decrease in antibiotic utilization via antimicrobial stewardship. Other authors have tended to report much more modest success. For instance, Holler et al.^17^ evaluated a multidisciplinary ASP at a Danish university hospital that incorporated prospective audit and feedback, prescriber education, and departmental prescribing reports. Despite implementation across multiple hospital departments, piperacillin-tazobactam use decreased by only 19.5% compared with baseline levels. Similarly, Wilson et al.^18^ described a resident-led quality improvement initiative at a single medical center in the USA targeting empiric piperacillin-tazobactam use for community-acquired intra-abdominal infections. Through educational interventions, treatment algorithms, and stewardship pharmacist support, piperacillin-tazobactam consumption decreased from 54.1 to 22.3 DOT per 1,000 patient-days, representing an approximately 59% reduction. Another example was published by Timbrook et al.^19^ using data from a single academic medical center where stewardship-driven formulary and guideline changes resulted in an overall 16% reduction in stewardship-targeted antibiotic utilization. Although substantial reductions were observed for fluoroquinolones (56%) and carbapenems (31%), decreases in piperacillin-tazobactam utilization were considerably smaller than those observed in our study.

Our strategy to discourage Pseudomonas coverage in low-risk patients led to the expected increase in the use of the recommended alternative antibiotic ceftriaxone by 67%. This was associated with a decrease in the susceptibility of *E. coli* to ceftriaxone with a nadir of 84% and in that of *K*. *pneumoniae* featured a nadir of 85%. Importantly, neither has gone lower than 80% susceptibility during the study period, which is our hospital’s cutoff for recommending discontinuation of first-line use of an antibiotic.

Unexpectedly, our study showed that the susceptibility to cefepime improved, in parallel with Piperacillin-tazobactam, from 77% to 96%, despite a 2.6-fold increase in cefepime utilization. This is a novel finding because to the best of our knowledge this association has never before been reported in the literature. The closest to our results regarding cefepime comes from Paladino et al.,^20^ where the researchers reported the converse relationship. Namely, they found that increased piperacillin-tazobactam consumption was associated with reduced cefepime susceptibility among *P. aeruginosa* and other Gram-negative pathogens, suggesting the presence of shared resistance mechanisms and cross-selection pressure. Our findings extend these observations by suggesting that removal of piperacillin-tazobactam selective pressure may facilitate recovery of cefepime susceptibility at the institutional level. We suspect our findings may be due to the shared modes of bacterial resistance that affect different beta-lactam antibiotics. The main genetic modes of adaptation in Pseudomonas sp. are inducible AmpC β-lactamase expression, efflux pump upregulation, porin loss, and acquisition of horizontally transferred resistance determinants.^21^ These mechanisms frequently confer resistance to multiple β-lactam agents simultaneously, resulting in complex cross-resistance phenotypes. Specifically, work by Livermore^22^ has demonstrated that AmpC-mediated resistance and permeability defects may affect susceptibility to both piperacillin-tazobactam and cefepime, suggesting that selective pressure from one agent may influence susceptibility to the other. This mechanistic framework may help explain our findings as well.

Traditional antimicrobial stewardship paradigms generally assume that increased use of a specific antibiotic will ultimately promote resistance to that agent. However, ecological studies have demonstrated that resistance emergence is often determined by the cumulative selective pressure exerted by multiple antimicrobial classes rather than by direct exposure alone.^23^ Our findings suggest that the reduction in piperacillin-tazobactam use may have removed a dominant source of selective pressure favoring multidrug-resistant *P. aeruginosa* strains, thereby allowing re-emergence of more susceptible populations. Under this hypothesis, piperacillin-tazobactam exposure may have contributed disproportionately to maintenance of resistance mechanisms that simultaneously impaired activity of both piperacillin-tazobactam and cefepime.

We must point out that several other aspects of the ASP intervention may have contributed to the observed outcomes. In addition to reducing piperacillin-tazobactam utilization, stewardship efforts promoted optimization of β-lactam pharmacodynamics through increased cefepime dosing and extended-infusion piperacillin-tazobactam and eventually extended-infusion cefepime administration. Pharmacokinetic-pharmacodynamic studies have consistently demonstrated that maximizing the time free drug concentrations remain above the minimum inhibitory concentration improves bacterial killing and may reduce the emergence of resistance^24,25^ with the associated clinical outcomes being recently reviewed by our group.^12^ Consequently, the observed improvements in susceptibility in our study likely reflect both reduced selective pressure and enhanced antimicrobial exposure resulting from stewardship-guided dosing optimization.

The measured increase in *E. cloacae* complex susceptibility to piperacillin-tazobactam in our study provides additional support for the impact of stewardship interventions on institutional resistance patterns. Nevertheless, the reader is likely aware that the 2024 IDSA guidelines for the treatment of multidrug-resistant Gram-negative bacteria^4^ recommend against the empiric use of Piperacillin-tazobactam for the treatment of most *E. cloacae* infections due to inducible chromosomal AmpC β-lactamases production.^26^ Therefore, our findings regarding Enterobacter carry mechanistic and stewardship importance but cannot be considered of clinical significance.

The results presented here could carry important implications for contemporary antimicrobial stewardship strategies. Historically, stewardship programs have emphasized carbapenem restriction to limit the emergence of carbapenem-resistant organisms. However, increasing evidence suggests that other broad-spectrum β-lactams such as piperacillin-tazobactam may also play substantial roles in resistance selection. Recent IDSA guidance highlights the importance of minimizing unnecessary antipseudomonal therapy and tailoring treatment according to local epidemiology and patient-specific risk factors.^4^ Our ASP approach closely follows these recommendations and the data we presented here emphasizes their appropriateness.

### Study limitations

It is important to note that our study has several limitations. First, it represents the experience of a single institution and may not be generalizable to settings with different patient populations, resistance mechanisms, or prescribing practices. Second, the observational design precludes definitive causal inference. Multiple stewardship interventions—including restriction of piperacillin-tazobactam use, increased cefepime utilization, cefepime dose optimization, and implementation of piperacillin-tazobactam extended-infusion therapy—were introduced concurrently, making it impossible to isolate the effect of any individual intervention. Third, the small number of annual observations limited statistical power and likely contributed to the inability to demonstrate significance for several strong correlation trends. Fourth, susceptibility data were derived from institutional antibiograms and therefore may be influenced by changes in isolate composition, patient mix, or local epidemiology. Finally, molecular characterization of resistance mechanisms was not performed, preventing direct assessment of the pathways underlying the observed susceptibility changes.

Despite these limitations, our study demonstrates that substantial improvements in susceptibility among *P. aeruginosa* and *E. cloacae* complex can occur following targeted stewardship interventions. The simultaneous restoration of susceptibility to both piperacillin-tazobactam and cefepime despite increased cefepime use is particularly compelling and suggests that resistance selection may be driven by shared ecological and mechanistic factors. Future multicenter studies incorporating molecular epidemiology, resistance mechanism analysis, and patient-level prescribing data are needed to confirm these findings and determine whether piperacillin-tazobactam functions as a key driver of β-lactam resistance in other healthcare settings.

In conclusion, an antimicrobial stewardship strategy centered on substantial reduction of piperacillin-tazobactam utilization was associated with marked improvements in susceptibility among *P. aeruginosa* and *E. cloacae* complex. The recovery of cefepime susceptibility despite increased cefepime use supports the importance of shared resistance mechanisms and raises the possibility that piperacillin-tazobactam exposure may exert a disproportionate influence on institutional β-lactam resistance patterns. These findings highlight the potential for targeted antimicrobial stewardship interventions to reverse resistance trends and preserve the effectiveness of critical antipseudomonal agents.

## Data Availability

The original data is available upon request.

## Table 1. Long description

A table detailing interventions by an Antimicrobial Stewardship Program from April to September 2023. The table has 10 rows and 2 columns. The first column lists the months and specific dates, and the second column describes the interventions. Row 1: April 2023, Piperacillin/tazobactam was changed to extended infusion dosing to enhance pharmacodynamic target attainment. Row 2: Cefepime was changed to 2 g from 1 g standard dosing to prevent development of resistance. Row 3: A prominent note was placed in ordering set stating: Piperacillin/Tazobactam is inadequate as empiric coverage for Enterobacter or Pseudomonas per Crouse 2023 Antibiogram. Row 4: Cefepime + metronidazole was substituted for piperacillin/tazobactam as the first-choice antibiotic in the relevant order sets. Row 5: Susceptibility to cefepime was unhidden culture results. Row 6: An email was sent from the hospital Chief of Medicine to all physicians highlighting the concerning antibiogram results and the above changes. Row 7: Daily (Monday through Friday) screens by the ASP pharmacist of all piperacillin/tazobactam orders and in-person evaluation by an ID physician of selected cases to consider change to cefepime if empiric Pseudomonas coverage is needed or de-escalation to ceftriaxone if empiric Pseudomonas coverage is not needed, followed by direct reach out by the ASP pharmacist, over phone or in person, to prescribers to deliver the appropriate antibiotic recommendations—this ASP activity continues to date. Row 8: ASP pharmacist presented the above recommendations in PowerPoint format at the monthly hospitalists’ meeting. Row 9: May 2023, Ninety paper notices were posted next to most hospital computer terminals summarizing the piperacillin/tazobactam to cefepime change for empiric Pseudomonas coverage. Row 10: June 2023, The ASP pharmacist sent FYI emails to all hospitalists and mid-level providers highlighting the above-listed recommendations regarding the preferential use of cefepime (plus metronidazole if anaerobic coverage needed) over Zosyn in suspected Pseudomonas infections and the preferred use of ceftriaxone over cefepime for Pseudomonas if empiric Pseudomonas coverage was not needed, followed by direct reach out by the ASP pharmacist, over phone or in person, to prescribers to deliver the appropriate antibiotic recommendations—this ASP activity continues to date. Row 11: The ASP pharmacist presented the above recommendations in PowerPoint format at the monthly physician assistants’ meeting. Row 12: The ASP pharmacist initiated daily (Monday through Friday) screens of all ceftriaxone orders followed by discussion with prescribers to encourage adherence to the preferred 2 g dosing regimen—this ASP activity continues to date. Row 13: September 2023, The ASP pharmacist presented the above recommendations in PowerPoint format at the monthly nurse practitioners’ meeting.

Navigate back to Table 1..

## Table 3. Long description

The table presents data on the correlation between antibiotic use and susceptibility for Pseudomonas aeruginosa and Enterobacter cloacae complex. It includes columns for antibiotic names, days of therapy per 1,000 patient days, annual susceptibility at time t + 1 year, Spearman’s correlation (ρ), P value (two-sided), and interpretation. The table is divided into sections for different antibiotics and bacteria. For Pseudomonas aeruginosa, antibiotics listed include piperacillin-tazobactam, cefepime, meropenem, ciprofloxacin, and levofloxacin. For Enterobacter cloacae complex, antibiotics listed include piperacillin-tazobactam, cefepime, meropenem, ertapenem, ciprofloxacin, and levofloxacin. Each section provides specific data points for days of therapy, susceptibility percentages, correlation values, P values, and interpretations of these correlations.

Navigate back to Table 3..
